# Adaptation of a social risk screening and referral initiative across clinical populations, settings, and contexts in the Department of Veterans Affairs Health System

**DOI:** 10.3389/frhs.2022.958969

**Published:** 2023-01-30

**Authors:** Alicia J. Cohen, Lauren E. Russell, A. Rani Elwy, Kathleen M. Mitchell, Portia Y. Cornell, Jennifer W. Silva, Ernest Moy, Meaghan A. Kennedy

**Affiliations:** ^1^ Center of Innovation in Long Term Services and Supports, VA Providence Healthcare System, Providence, RI, United States; ^2^ Department of Family Medicine, Warren Alpert Medical School, Brown University, Providence, RI, United States; ^3^ Department of Health Services, Policy, and Practice, School of Public Health, Brown University, Providence, RI, United States; ^4^ Office of Health Equity, Veterans Health Administration, Washington, DC, United States; ^5^ Center for Healthcare Organization and Implementation Research, VA Bedford Healthcare System, Bedford, MA, United States; ^6^ Department of Psychiatry and Human Behavior, Warren Alpert Medical School, Brown University, Providence, RI, United States; ^7^ New England Geriatric Research, Education, and Clinical Center, VA Bedford Healthcare System, Bedford, MA, United States; ^8^ Department of Veterans Affairs, National Social Work Program Office, Care Management and Social Work, Patient Care Services, Washington, DC, United States; ^9^ Department of Family Medicine, Boston University School of Medicine, Boston, MA, United States; ^10^ Department of Community and Family Medicine, Geisel School of Medicine at Dartmouth, Hanover, NH, United States

**Keywords:** social determinants of health (SDOH), social risks, social needs, veterans, adaptation, implementation science, health equity

## Abstract

Identifying and addressing social risks and social needs in healthcare settings is an important step towards achieving health equity. Assessing Circumstances and Offering Resources for Needs (ACORN) is a Department of Veterans Affairs (VA) social risk screening and referral model that aims to systematically identify and address social needs. Since initial piloting in 2018, our team has collaborated with clinical and operations partners to implement ACORN across multiple VA clinical settings while adapting and tailoring the initiative to meet the needs of different populations, specialties, and individuals administering screening. Given ACORN's complexity as a growing initiative with multiple partners and frequent real-time modifications within a large national healthcare system, we recognized a need to systematically document the rationale and process of adaptations over time. We looked to three implementation frameworks—RE-AIM, the Adaptome, and FRAME—to describe the rationale for adaptations, the nature of and context within which adaptations were made, and the details of each adaptation. In this manuscript, we uniquely interweave these three frameworks to document adaptations to ACORN across diverse VA clinical settings, with a focus on how adaptations support the promotion of heath equity in the Veteran population.

## Introduction

Social risks and social needs—specific adverse social conditions such as unstable housing or food insecurity, and an individual's perceived and prioritized needs—are associated with negative health outcomes throughout the lifespan (1–4). Addressing social needs, which are often rooted in underlying societal inequities and systemic racism, is critical to advancing health equity. The National Academies of Sciences, Engineering, and Medicine, Centers for Medicare & Medicaid Services, and other professional organizations and payers have all called for improved integration of social care into medical care (5–9). While systematic screening for social risks and interventions to address social needs are increasingly being implemented in health care settings, there is limited evidence regarding best practices (10–12). As the nation's largest integrated healthcare system with a robust network of embedded social services, the Department of Veterans Affairs health system (VA) is uniquely positioned to address social needs. Although the VA currently has universal screening for housing instability (13), food insecurity (14), and intimate partner violence (15), VA does not yet systematically screen Veterans for social risks more broadly. Given the medical and social complexity that many Veterans experience (4, 16), a comprehensive approach to identifying and addressing social risks is needed.

Assessing Circumstances and Offering Resources for Needs (ACORN) is a quality improvement initiative conducted in partnership with the VA Office of Health Equity and VA National Social Work Program, Care Management and Social Work Services to systematically identify and address Veterans' social risks and social needs (17). Our overall aim is to implement and evaluate ACORN to support systematic screening of all Veterans, improve health outcomes, and advance health equity by providing Veterans resources and referrals that meet their individual needs. First developed and piloted in 2018 in the VA New England Healthcare System, ACORN is broadly based on several well-established, evidence-based social risk screening and referral models, including a number that have been successfully implemented in other large healthcare systems (7, 18–23). These types of models are widely used and have been shown to improve identification of needs and successful connection of patients with resources. There is also an emerging evidence base demonstrating the impact of these models on improved health and decreased acute care utilization (20, 22, 23). As our team developed the core ACORN model, we aimed to integrate existing VA universal screening processes with essential features of evidence-based social risk screening and referral programs and expert guidelines (e.g., key social risk screening domains, validated and/or widely used screening questions, and resource guides). Following development and successful pilots, the ACORN model has been iteratively adapted to meet the unique needs and context of different Veteran populations, clinical specialties, and VA settings.

Given frequent real-time adaptation involving multiple partners as well as rapid dissemination, we have recognized a critical need to systematically document program adaptations over time to both understand the rationale for modifications and rigorously plan our future directions (24, 25). Implementation frameworks offer a systematic approach to succinctly and thoroughly summarize and assess the impact of adaptations (25–29). In this manuscript, we use ACORN as a case study to demonstrate a novel interweaving of three implementation frameworks—the Reach, Effectiveness, Adoption, Implementation, Maintenance (RE-AIM) framework (26, 27), the Adaptome (25), and FRAME (Framework for Reporting Adaptations and Modifications-Enhanced) (29)—to: (1) describe the process of adapting ACORN across piloting, implementation, and scale up phases; (2) summarize the rationale, nature, and components of ACORN adaptations; and (3) describe future directions for this work**.**

## Materials and methods

### Establishing core components of ACORN

Core components of the ACORN initiative include: (1) administration of the standardized ACORN social risk screener; (2) provision of resource guides and referrals to VA and community services for any identified needs; and (3) a mechanism to address urgent needs at the time of screening. An interprofessional team of physicians, clinical psychologists, social workers, clinical informaticists, and health services researchers developed the original ACORN screener, which covered nine social risk domains: housing instability, food insecurity, utility needs, lack of transportation, social isolation/loneliness, interpersonal violence, legal assistance, educational needs, and employment concerns. We selected domains that were recommended by key health care and policy organizations (5, 7) and could reasonably be addressed through available VA or community resources. All decisions were informed by multi-partner feedback during pre-implementation planning and initial piloting, including feedback from Veterans. We deliberately included VA's universal screening questions for housing instability and food insecurity to align existing screening efforts. Additional screening items included both existing questions from commonly used social risk screening instruments (7, 18, 19), as well as new questions specific to Veterans' needs developed by the interprofessional ACORN team. Gaps that were identified in existing VA screening protocols for housing instability and food insecurity—in particular, lack of a screening question that explicitly assessed current or urgent needs—informed development of ACORN questions assessing current or urgent needs related to food, housing, transportation, and utilities. We then further refined the screener through cognitive testing with Veterans. A trained interviewer inserted additional questions into a well-developed draft of the screener to prompt discussion of Veterans' comprehension of the questions and response options as well as their comfort answering questions related to the nine domains. Revisions were made based on feedback from cognitive testing to ensure questions were Veteran-centric and at an appropriate level of health literacy to understand and respond. The newly revised version was then field tested and finalized.

In consultation with VA leaders and subject matter experts involved in the development and implementation of VA's existing social risk screening and follow up processes, we incorporated VA's already well-defined referral pathways for housing instability and food insecurity into the ACORN initiative. These established pathways as well as that for the VA's intimate partner violence screening also informed ACORN follow up processes for other screening domains. To address any identified needs, VA staff provide Veterans who screen positive with relevant resources and referrals. Depending on the clinical setting, this may include providing Veterans with resource guides for specific social risk domains, referring Veterans to VA or community resources, and/or providing navigation support to access programs and services. ACORN resource guides are curated, one-page lists of high-quality VA and non-VA programs and services tailored to local communities for each of the social risk domains covered in the ACORN screener (30). Resource guides are given to Veterans who screen positive for one or more needs by VA staff, typically in conjunction with other interventions (e.g., referrals to social work or other services). We intentionally created room for variation in how positive screens are addressed based on who is conducting the screening and in what context. However, there are certain urgent social risks (e.g., safety concerns, currently unhoused, inadequate food for the week, or utilities shut off) which generally warrant immediate action, so we also ensure a mechanism is in place at each site to provide a “warm handoff” to a social worker (if staff other than a social worker are implementing ACORN) or otherwise address urgent needs.

We initially pilot tested ACORN in an outpatient mental health clinic within a suburban New England VA Medical Center. Veterans completed the screener in the waiting room upon arriving for their clinic visits, a process that leveraged existing workflows for pre-visit clinical screening in that setting. The clinical team reviewed screening results, gave Veterans screening positive information about VA and community resources, and referred them to social work or other relevant specialties when appropriate. Our evaluation of the initial pilot, which included formal data collection as well as informal feedback gleaned from regular meetings with staff, assessed: (1) the feasibility of implementing ACORN in this setting; (2) prevalence of reported social risks; (3) Veteran and staff reported acceptability, appropriateness, and perceived importance of screening for social risks; and (4) Veterans' use of and opinion regarding resource guides.

### Adapting and tailoring ACORN to meet contextual demands

We applied lessons learned from the initial pilot as we iteratively adapted and tailored ACORN to diverse clinical settings (outpatient, inpatient, emergency department), specialties (general primary care, women's health, social work), and individuals administering screening (Veterans, nurses, social workers, Peer Specialists). Our team receives feedback from the field on a regular basis and adapts to optimize ACORN in collaboration with clinical teams. We have adapted ACORN to maximize the number and range of Veterans screened, impact of the program, and scalability over time. In addition to planned adaptations, we have made unplanned adaptations such as those necessitated by the COVID-19 pandemic (e.g., creating an option for virtual screening). In all phases, an interprofessional implementation and evaluation team has worked with frontline staff to optimize ACORN with respect to existing clinical workflows and preferences.

### Integration of frameworks to document and describe adaptations

**Figure 1:**
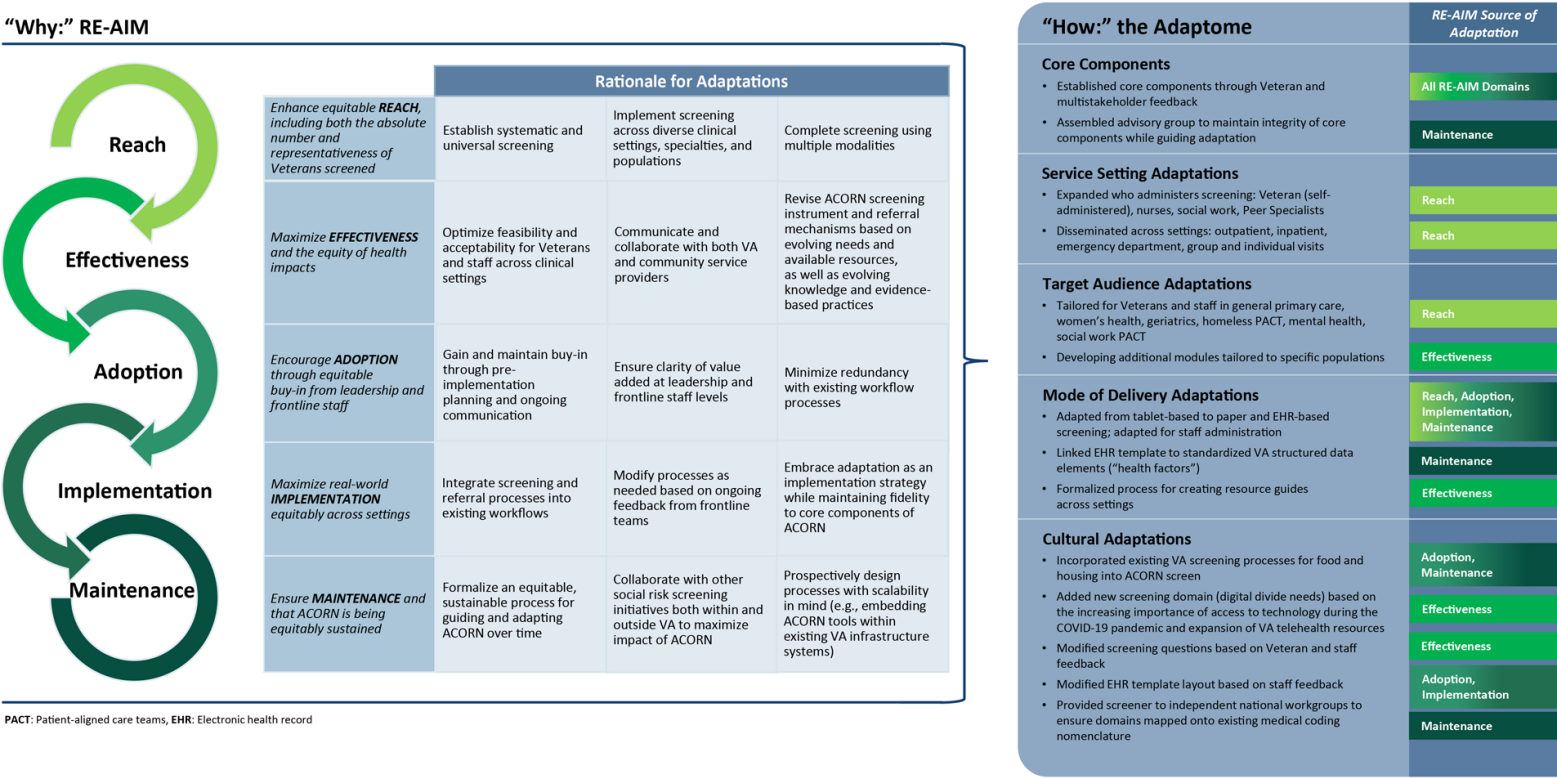
Summary of the rationale (“why”) for adaptations to the ACORN initiative using the RE-AIM framework [Cites are 26, 31, 33], mapped to the nature (“how”) of adaptations using the Adaptome [cite is 25].

We selected RE-AIM, the Adaptome, and FRAME to document and describe ACORN initiative adaptations because of their wide use and applicability to implementation of complex interventions across all phases from pre-implementation planning to evaluation (25–27, 29, 31–34). We first summarized the rationale for the need for adaptations (“*why*”) using RE-AIM. We then categorized the nature of adaptations (“*how*”) by Adaptome domain (core components, service setting, target audience, mode of delivery, cultural) (25) and mapped corresponding RE-AIM domains for each adaptation onto the Adaptome. This mapping allowed us to demonstrate the interrelationship between the “*why*” and the “*how*,” which we visually highlighted through the creation of an integrated figure (Figure 1). Finally, we used FRAME, organized by nature of adaptation laid out in the Adaptome, to document in more detail examples of key planned and unplanned adaptations (Supplementary Table 1)*.* FRAME elements selected for our final table (what was modified, when, planned/unplanned, who decided, level of delivery, nature of modifications, and reasons) were those that were most salient to our initiative.

## Results

### Rationale for adaptations: RE-AIM provides the “why” for adaptations

RE-AIM is a framework that is widely used to plan programs, evaluate their implementation, and assess their potential for translation into practice. RE-AIM is based on five key outcome domains: REACH (who is the target population and who ultimately receives the intervention), EFFECTIVENESS (impact of the initiative on desired outcomes and the likelihood of negative outcomes), ADOPTION (who is initiating the intervention and where), IMPLEMENTATION (fidelity to the intervention protocol and any adaptations), and MAINTENANCE (the extent to which the intervention becomes institutionalized and sustained) (26, 31). RE-AIM has also been used to systematically document and assess adaptations at all stages of program implementation (27, 32, 34). In this work, we use a more recent, explicit emphasis on health equity as a fundamental element that needs to be addressed across all RE-AIM dimensions to guide documentation of the rationale for each adaptation (33) (Figure 1).

#### Reach

To enhance the equitable reach of ACORN including both the absolute number and representativeness of Veterans screened, we adapted it to support systematic, universal screening for all Veterans. These adaptations included implementing screening across diverse clinical settings, specialties, and populations, and screening using multiple modalities [e.g., paper, electronic tablet, and administered directly in the electronic health record (EHR)]. We have explicitly sought to adapt ACORN to populations that are at particularly high risk for experiencing social risks and health disparities.

ACORN was originally designed to be administered in the outpatient setting to systematically screen Veterans for social risks that might otherwise go unidentified. The decision to initially pilot ACORN in an outpatient mental health clinic was largely pragmatic—the ACORN screener was designed for Veterans to self-administer in the waiting room using a VA-developed tablet-based screening platform (eScreening) that synchronized with the electronic health record (35), and a mental health clinic at our pilot site was already using this platform for other clinical assessments. This pre-existing infrastructure and staff familiarity with eScreening increased staff buy-in for ACORN and allowed for easier integration of the ACORN screener into existing workflows. We subsequently expanded to other outpatient settings including general primary care as well as specialty clinics such as women's health, geriatrics, and a primary care clinic for Veterans experiencing homelessness.

A major reach-focused adaptation entailed creating an option for staff to administer the ACORN screener in lieu of Veterans completing screening on their own in the waiting room. Initially developed in response to the COVID-19 pandemic when in-person visits were temporarily halted—and subsequently when there were infection control concerns with having electronic tablets for shared patient use in the waiting room—we created an option for staff to administer ACORN and enter screening results directly in the EHR. While this was an unplanned adaptation made rapidly and out of necessity, providing an EHR-based option for staff-administered screening has greatly facilitated our ability to scale ACORN to other settings and populations.

Additionally, in order to allow ACORN to be administered across as many settings as possible and provide flexibility based on local staffing and workflows, we have developed adaptations in which the screener can be administered by a range of clinical staff including nurses and social workers, as well as non-clinical staff such as Peer Specialists.

Recognizing the different touchpoints Veterans have with the health care system, and particularly that acute care visits may provide an opportunity to screen Veterans who have not otherwise presented for outpatient care, we are currently adapting ACORN for administration in inpatient, emergency department (ED), and urgent care settings. Veterans presenting to the ED or being hospitalized may also be at particularly high risk for experiencing unmet social needs (36), making it crucial to screen this patient population to equitably expand ACORN's reach. We are also developing adaptations in which ACORN can be administered during group visits such as advanced care planning groups and group health coaching.

#### Effectiveness

To maximize effectiveness as well as the equity of health impacts, adaptations focused on: (a) screening and referral processes that were feasible, acceptable, and appropriate for Veterans and clinical staff across a range of settings; and (b) optimizing communication and collaboration with both VA and non-VA service providers. Whether screening is Veteran self-administered or staff-administered, in each instance we have worked to create setting and specialty-specific workflows around how and when to best introduce ACORN, and to ensure that Veterans with identified needs receive appropriate resources and referrals. As an example, nurses within the VA can either place a formal consult to a social worker for case management or do a warm handoff to either a social worker or another clinician. Peers, however, are unable to place formal consults, but can complete warm handoffs, provide community referrals, or otherwise work with Veterans directly to try to help address certain social risks. When ACORN is implemented in a group visit setting (still in planning stages), Veterans will likely self-administer the screener and then receive follow up with a social worker after the group to address any identified needs.

We have also sought to improve effectiveness and support equity by soliciting feedback from both Veterans and ACORN clinical and operations partners for all adaptations, both planned and unplanned, during regular check-in meetings with various partners and pilot sites. We have been conducting follow up surveys and interviews with both Veterans and staff after ACORN has been implemented in a new context. When considering additional screening questions or domains, we have ensured partner feedback at all stages of development from determining relevance of the questions, to developing initial wording, to refining wording based on cognitive interviews with Veterans, to subsequent formal piloting/field testing.

As part of planned future effectiveness-related adaptations, we are working on formalizing systems for closed-loop communication to determine if a Veteran was able to successfully connect with recommended resources and if their needs have been adequately met. We have developed an “ACORN Follow-Up” template for the EHR that includes an assessment of which needs have been met, any barriers encountered in accessing resources, and any remaining needs. However, this has not yet been widely implemented or tested across sites and setting- and specialty-specific workflows are still in development.

#### Adoption

Adoption-focused adaptations aimed to gain and maintain equitable buy-in from both leadership and frontline staff in each clinical setting through pre-implementation planning and regular check-ins. Successful adoption across settings hinged on establishing the value of ACORN both for clinical and non-clinical specialties without a prior mechanism for systematic social risk screening as well as for those already engaged in some degree of social risk screening. As an example of the latter, while VA social workers routinely conduct comprehensive biopsychosocial assessments when working with Veterans, the VA National Social Work Program was interested in potential applications of the ACORN screener as an initial triage assessment tool for social workers in the primary care setting. We worked with Social Work leadership and staff during planning meetings both to explore the benefits of ACORN in this context and to minimize any perceived redundancy with current clinical processes among frontline staff. We have found pre-implementation planning with both leadership and frontline staff to be essential for initial buy-in and subsequent adoption of the intervention across service settings.

#### Implementation

To maximize real-world implementation equitably across settings, adaptations were made to ensure screening and referral processes were integrated into existing workflows and that lower-resource settings such as VA community-based outpatient clinics and/or rural sites that may have fewer onsite resources have the necessary support to effectively implement ACORN and address identified needs. We also sought continuous feedback from frontline teams both *ad hoc* and through regularly scheduled meetings to modify procedures in ways that embraced adaptation as an implementation strategy while also facilitating fidelity to core components across sites. This feedback has resulted in real-time adjustments which have supported successful implementation at sites and promoted innovation and further adaptation. For example, in our ongoing work with Peer Specialists, both Peers and clinical leadership suggested that we expand ACORN screening from the outpatient setting to an inpatient psychiatric unit. We then explored this suggestion with the psychiatric unit frontline staff and collaboratively developed a workflow which we are beginning to implement in that setting. In our work with the VA National Social Work Program, in which we are implementing a social worker-administered adaptation of ACORN at 11 different clinical sites, we have regular all-site meetings to share experiences, lessons learned, barriers encountered, and potential solutions as well as to provide technical and administrative support to sites. This learning community approach has been highly valuable to both ACORN leadership and individual sites and is an approach we plan to continue in future multi-site implementations.

Additional implementation-related adaptations included incorporating a formal “disposition” section indicating what follow-up actions were taken (e.g., referral to social work or other specialties such as mental health, provision of specific resource guides, referrals to community organizations, any follow-up appointments scheduled, etc.), as well as embedding free-text fields into the standardized ACORN EHR template. These adaptations allowed staff administering the screener to maintain fidelity to the core elements of ACORN and easily document actions taken, while also having space to include additional notes to maximize the clinical usefulness of the template. Another implementation adaptation consisted of providing laminated and paper copies of a “clipboard” version of the screener for times when ACORN is administered by staff when they are not immediately in front of a computer. Providing a clipboard version of the screener enabled staff to administer the screener in a broader range of clinical contexts and maintain fidelity to the wording of the questions rather than trying to paraphrase based on memory.

#### Maintenance

Lastly, to ensure maintenance, we are formalizing an equitable, sustainable process for guiding and adapting ACORN over time—specifically, convening an interprofessional ACORN Partner Engagement Group with representation from key VA operational offices, other researchers engaged in social determinants of health-related work, and Veteran representatives to provide subject matter expertise and feedback on proposed developments or changes. The objectives of this group are to ensure ACORN initiatives are designed and implemented with a health equity lens, align ACORN efforts with partner office priorities and clinical workflows, and maintain integrity of core components while helping guide larger changes.

Additionally, we have built structured data capture elements into the ACORN EHR template, enabling screening responses and follow-up actions to be tracked in the VA administrative and clinical database to support evaluation efforts and scalability. In order to maximize the impact, relevance, and sustainability of ACORN, we have engaged in ongoing dialogue and collaboration with other teams engaged in health equity and social risk-related research and quality improvement initiatives within VA. Outside of the VA, we have coordinated with national entities engaged in work around assessing, documenting, and addressing social risks and social needs to optimize the interoperability of screening tools and resulting diagnostic coding for electronic health information exchange.

### Nature of adaptations: Adaptome provides the “how” for adaptations

After establishing *why* each of these adaptations was needed using RE-AIM, we used the Adaptome, which provides a framework for establishing core components of an intervention as well as characterizing types of adaptations within and across various contexts, to describe *how* core components of ACORN were determined and summarize the nature of adaptations made (25). We then mapped each of the adaptations catalogued in the Adaptome to corresponding RE-AIM constructs to show the interrelationship between the “*why*” and the “*how*” (Figure 1).

#### Service setting adaptations

Adaptations to the service setting in which ACORN is implemented have included both who administered the screening (e.g., Veteran, nurse, social worker, Peer Specialist) and clinical setting (e.g., outpatient, inpatient, ED). Additional service setting adaptations currently underway include exploration of use during group visits and in urgent care settings, as well as administration by patient navigators. Each of these adaptations has required careful consideration of workflows and how different staff interact with Veterans in various settings (e.g., paper vs. electronic administration, remote vs. in-person, and whether ACORN screening is conducted alone or as part of other assessments). Nurses in the outpatient setting, for example, often administer ACORN during a pre-visit intake along with other routine clinical screening questions. They are then able to provide resource guides, as relevant, and depending on local workflows either place needed consults, initiate needed warm handoffs, or alert the clinician seeing the Veteran to provide needed follow up. Social workers implementing ACORN, whether in the outpatient or acute care setting, typically administer ACORN screening as part of an initial triage assessment, and based on screening results as well as current clinical demands, either follow-up with a full biopsychosocial assessment or triage any acute needs in the moment and arrange for a more comprehensive assessment at a later date. When Peers Specialists are using ACORN, the screener may be administered alone or as part of other interventions they are trained to implement [e.g., VA's Whole Health programs (37)]. Peers also determine which identified needs they can help a Veteran navigate on their own through the provision of resources and referrals vs. needs that would be better addressed through a warm handoff to a social worker, mental health provider, or the Veteran's primary care provider.

#### Target audience adaptations

We have made several adaptations to ACORN focused on the target audience, including both modifications to the clinical specialty where screening was administered (general primary care, women's health, geriatrics, homeless clinic, mental health, and social work) and the development of additional screening questions and resources tailored to specific populations (e.g., Veterans experiencing homelessness, older adults). As an example, in partnership with researchers and clinicians at a VA primary care clinic for Veterans experiencing homelessness, we modified the ACORN screener to meet the environmental context and specific needs of this population. Adaptions to the screener included the incorporation of two questions pertaining to where Veterans have stayed over the past month and where they slept the previous night, as well as the exclusion of the utilities domain. Based on feedback from subject matter experts and Veterans experiencing homelessness, our team eliminated the utilities domain because it would not pertain to a substantial proportion this population, such as those residing in shelters or congregate living facilities, cars, tents, or on the street.

#### Mode of delivery adaptations

Key adaptations to delivery modality have included adapting the initial tablet-based screening process for administration on paper and via the EHR, as well as shifting from Veteran self-administration to clinical staff-administration so that screening could be conducted either in-person or via telehealth, which was critical with the onset of the COVID-19 pandemic. We worked with VA programmers to create a universal EHR template that could be easily imported at clinical sites nationally, and made iterative modifications to ensure the template was flexible enough to be useful across clinical settings. We further mapped all structured fields in the template to standardized data elements (“health factors”) in the EHR, which has allowed us to easily extract these data for evaluation purposes, as well as to link ACORN screening data to relevant sociodemographic, clinical, and administrative data in the Corporate Data Warehouse, a VA data platform that serves as a national repository of EHR data from VA clinical and administrative systems (38). Finally, we formalized a process for the creation of geographically tailored resource guides that can be used as a cross-cutting tool for both Veterans and staff across a range of settings and specialties. This process has included the development of a “how-to” ACORN Resource Guide Manual with resource guide templates containing both standardized and setting-specific customizable elements that are also, by design, tailorable to local needs and contexts (30).

#### Cultural adaptations

Cultural adaptations were made to ensure ACORN aligned with the needs and preferences of specific teams and settings as well existing efforts both within and outside the VA. For example, we incorporated existing VA universal screening processes for food insecurity and housing instability into the ACORN screening tool so that when ACORN is administered in the EHR, it also satisfies and “checks off” these VA screening requirements. We also modified several aspects of the screener itself, including: (1) adjusting the wording of certain screening questions based on Veteran and partner feedback; (2) modifying aspects of the EHR template based on staff feedback such as adding free text fields for further documentation of relevant clinical information; and (3) changing the layout of the Veteran self-administered paper version of the screener so that it is easier for staff to visually scan for positive responses. We added a new screening domain related to technology, phone, and internet accessbased on the increasing importance of access to technology during the COVID-19 pandemic and expansion of VA telehealth resources. We also removed the interpersonal violence domain from the screener in certain settings (in one instance because of perceived redundancy with existing screening questions, and in other instances due to concerns about availability of immediate follow-up). Finally, we provided the ACORN screener to independent national workgroups to ensure each of the questions and domains mapped onto existing medical coding nomenclature [e.g., International Classification of Disease (ICD) codes].

### Detailed documentation of adaptations: FRAME

Finally, we used FRAME to document key planned and unplanned adaptations in more detail (Supplementary Table 1), categorized by Adaptome domain*.* Primary aspects documented based on FRAME include what was modified (content, evaluation, training, or context); when during the implementation process the modification was made; whether the adaptation was planned/proactive or unplanned/reactive; who decided to implement the adaptation; level of delivery (for whom the adaptation was made); nature of the adaptation (including tailoring, adding, removing, or substituting elements); and reasons for the adaptation including both the goal and relevant contextual factors (29).

An example of a context-related mode of delivery adaptation included pivoting from Veteran self-screening to staff-administered screening directly in the EHR (“what was modified”). We rapidly developed this adaptation during the ACORN pilot phase in response to the COVID-19 pandemic (“when,” “unplanned”) because there was a dramatic increase in telehealth and relying on in-person administration of the screener was not feasible (“contextual factors”). This adaptation was jointly developed by the core project team and frontline nurses at the pilot sites (“who decided”) in order to increase reach (“goal”) by allowing nurses to start administering the screener during virtual (telehealth and phone) visits (“level of delivery”). Subsequent planned adaptations related to this mode of delivery adaptation included linking the screening responses to structured data fields in the EHR so that the data could be more easily retrieved and analyzed for evaluation, and creating workflows that allowed the staff-administered ACORN screener to satisfy existing screening requirements within the VA for food insecurity and housing instability.

A content-related cultural adaptation included adding a new screening domain to the core screener to assess access to technology such as cell phone, computer, and internet (“what was modified,” “level of delivery”). We developed this adaptation in collaboration with VA operational partners (“who decided”) in 2021 during our implementation phase (“when”) following development of new processes in VA to address digital needs (“planned”). Our “goal” was to increase the clinical effectiveness and appropriateness of the screening tool given both increasing needs for technology access during the pandemic, as well as newly available resources in the VA to address these needs (“contextual factors”).

In ongoing work, we will continue to use FRAME to prospectively document and track both ACORN-wide adaptations as well as adaptations specific to individual sites and settings. Moving forward, we are considering adding an additional domain related to adaptation outcomes, both positive and negative. While each of the adaptations currently described were developed by or in collaboration with the ACORN team, as ACORN continues to be scaled and we find sites are initiating their own modifications, we will also start tracking the extent to which these modifications are fidelity consistent with the core components of ACORN. Current processes for documenting and tracking adaptations have included detailed notetaking during all meetings including regular check-ins with partners and pilot sites, as well as shared documents in Microsoft Teams.

## Discussion

We have involved a collaborative, interprofessional team with ongoing input from frontline staff, VA operational partners, and Veterans to iteratively adapt ACORN to a range of clinical settings and contexts. Key planned and unplanned adaptations spanned various practice settings, patient populations, modes of administration, and evolution of the social risk screener content. Documentation of these diverse adaptations has been particularly helpful to our team given the complexity of ACORN as a quality improvement initiative with multiple clinical, operations, and research partners in a large national healthcare system with geographically and programmatically distinct clinical settings, interprofessional teams, and innovative approaches to care delivery.

Rigorous documentation of adaptations over the lifecycle of an intervention is critical to understanding and optimizing implementation across populations, settings, and contexts (25, 27–29). Multiple implementation frameworks are often used in combination to leverage complementary content, and several combinations have been formally described in the literature (34, 39, 40). However, few are specifically focused on adaptation. One prominent example of an adaptation-focused blending of frameworks is work by Rabin and colleagues in which they used FRAME supplemented with additional elements from RE-AIM to assess adaptations across four different health system interventions (34). In this manuscript, we present a novel integration of RE-AIM, the Adaptome, and FRAME to systematically document and assess adaptations made across multiple complex pilots in real-world clinical settings. In our ongoing implementation and scale up efforts as well as future work, we are using these frameworks prospectively to document adaptations and adaptation outcomes as a key component of our planning and evaluative work.

Exploring the connection between these three frameworks has allowed us to think through and record the evolution of establishing core components of ACORN, rationale for why we initiated specific adaptations, how we made the adaptations, and the broader context in which they were made, as well as to create a detailed catalogue of the individual elements of each adaptation, however large or small. Visually mapping these frameworks has also provided an appreciation for multifaceted ways in which these various components are interrelated. For those adaptations where we applied these frameworks retrospectively, this process has helped us to better understand the nature of the adaptations made. As an example, while our adaptations have spanned service settings, target audiences, modes of delivery, and cultural adaptations, through the process of documenting and mapping our adaptations we realized that most of our adaptations to date have been related to context. Applying these frameworks prospectively to ongoing adaptations has helped us to both identify patterns from prior adaptations and identify potential blind spots or gaps that we can proactively address, particularly as they relate to promoting health equity.

This method of framework integration has several limitations. As with any framework, it can be difficult to categorize and succinctly distill complex adaptations. Additionally, although interweaving RE-AIM, the Adaptome, and FRAME provides a cohesive scaffolding for documenting the “why,” “how,” and “what” of adaptations, there is variation in terminology across frameworks that may need to be reconciled. Finally, the figures and tables can be complicated, particularly with large multi-site studies, and the FRAME table specifically has the potential to become unwieldy. When using this method, it is important to discuss as a team how best to operationalize and maintain the integrated frameworks for application in practice (e.g., collectively reviewing all additions on a regular basis to synthesize changes and share updates with key partners).

Social care interventions, by necessity, must be designed, implemented, and evaluated with an equity lens. As there are increasingly calls for the explicit integration of health equity into implementation science (41–44), there is also an emerging literature focused on the importance of health equity specifically as it relates to adaptation (33, 45). Furthermore, Baumann and colleagues speak to the importance of using adaptation not only to modify interventions, but also to modify implementation strategies as a critical component of addressing health inequities. Next steps for our team include exploring and tracking adaptations to implementation strategies (i.e., strategies specifically used to increase the uptake and dissemination of ACORN across settings), and particularly how these adaptations do or do not promote health equity. Future effectiveness evaluations will also examine the extent to which social risks are identified that would not otherwise have been routinely screened for during usual care across various populations and settings; potential differences in reported needs with Veteran self-administered vs. staff-administered screening and how this may vary by clinical specialty administering the screening; as well as changes in care processes (e.g., resources delivered, referrals made) to address unmet needs and the extent to which these changes are occurring in an equitable manner.

This article is the first we are aware of to use implementation frameworks to systematically document and track prior and ongoing adaptations across all stages of a social risk screening and referral intervention. Similar contributions from social care interventions across different health care settings are needed to collectively inform equitable best practices and policy.

## Data Availability

The original contributions presented in the study are included in the article/Supplementary Material, further inquiries can be directed to the corresponding author/s.
