# Supplementary material for: Adaptation of a social risk screening and referral initiative across clinical populations, settings, and contexts in the Department of Veterans Affairs Health System
Source: Front Health Serv. 2023 Jan 30;2:958969. doi: 10.3389/frhs.2022.958969 (PMC10012714; doi:10.3389/frhs.2022.958969)
Supplement: Supplementary file 1 [file Table1.docx]

**Table 1: Detailed Documentation of Adaptations Using FRAME^1^ , categorized by** **Adaptome domain^2^**

|  | **What was Modified** | **When** | **Planned/ Unplanned** | **Who Decided** | **Level of Delivery** | **Nature of Modifications** | **Reasons** |
| --- | --- | --- | --- | --- | --- | --- | --- |
| **Adaptome – Service Setting Adaptations** Allowed ACORN to be disseminated in a broader range of settings; provided flexibility around when and how ACORN is administered | | | | | | | |
| *Expanded WHO can administer the ACORN screener* | | | | | | | |
| ***Context***  ***(Personnel)*** | Shifted from Veteran self-administered screening to nurse-administered screening | 2020 at onset of COVID-19 pandemic  (pilot phase) | Unplanned/ reactive | ACORN Team and frontline clinical nurses | Clinician/ staff-level | Substitution  (who administers) | **Goal:** Increase reach  **Contextual factors:**   - VA nurses are trained to administer clinical screeners - Veteran self-screening not feasible at onset of pandemic due to shift to primarily telehealth care - Training nurses to use ACORN permitted  in-person and virtual implementation |
|  | Screening administered by social workers | 2021  (implementation phase) | Planned/ proactive | ACORN Team and National Social Work Program | Clinician/ staff-level | Substitution  (who administers) | **Goal:** Increase reach  **Contextual factors:**   - VA social workers routinely screen for social risks and needs - ACORN offered opportunity to implement a standardized social work triage intake assessment tool |
|  | Screening administered by Peer Specialists | 2021  (implementation phase) | Planned/ proactive | ACORN Team with Peer leadership, Peers | Clinician/ staff-level | Substitution  (who administers) | **Goal:** Increase reach; optimize acceptability; increase effectiveness; increase health equity  **Contextual factors:**   - Early pilots showed that some Veterans may benefit from more hands-on resource navigation support - Peers have expertise in community/VA resources, resource navigation, and serving as liaison to services - Unique relationship of Peers with Veterans via lived experience may increase reach and acceptability to vulnerable populations |
|  | **What was Modified** | **When** | **Planned/ Unplanned** | **Who Decided** | **Level of Delivery** | **Nature of Modifications** | **Reasons** |
|  | Screening administered by pharmacists | 2022  (scale up phase – in progress) | Planned/ proactive | Clinical pharmacist champion in collaboration with ACORN Team | Clinician/ staff-level | Substitution (who administers) | **Goal:** Increase reach  **Contextual factors:**   - VA clinical pharmacists serve in direct patient care roles, often seeing patients with complex needs (e.g., poorly controlled diabetes or heart failure) |
| *Expanded SETTINGS in which ACORN is implemented* | | | | | | | |
| ***Context***  ***(Setting)*** | Expanded from mental health to primary care | 2019  (pilot phase) | Planned/ proactive | ACORN Team, primary care clinical leadership, frontline nurse leadership, and regional (VISN) leadership | Clinic-level | Substitution (where administered) | **Goal:** Increase reach; increase health equity  **Contextual factors:**   - Successful piloting in a mental health clinic with lower patient volume led to greater comfort with expansion to primary care settings providing care to a larger number of patients - Standardized screening instruments are integrated into VA primary care workflows, staff are comfortable using them - VA primary care is organized into interprofessional teams (PACTs) that can help address unmet needs - Primary care sees patients regularly (generally at least yearly) offering an opportunity to systematize screening for all Veterans and increase reach for Veterans who may not already be connected with social work |
|  | Expanding to specialty clinics | 2022  (scale up phase – in progress) | Planned/ proactive | ACORN Team (informed by interest expressed by various local specialty clinics) | Clinic-level | Substitution (where administered) | **Goal:** increase reach; increase health equity  **Contextual factors:**   - Some VA facilities offer sub-specialty clinics for Veterans with specific conditions  (e.g., diabetes, heart failure) - Expanding to specialty clinics may increase reach to Veterans with complex health and social needs |
|  | **What was Modified** | **When** | **Planned/ Unplanned** | **Who Decided** | **Level of Delivery** | **Nature of Modifications** | **Reasons** |
|  | Expanding from outpatient to inpatient settings | 2022  (scale up phase – in progress) | Planned/ proactive | ACORN Team (informed by interested expressed by local inpatient service) | Unit-level | Substitution (where administered) | **Goal:** increase reach; increase health equity  **Contextual factors:**   - Inpatient setting may present a unique opportunity to screen patients who might not otherwise present for outpatient care - Addressing unmet social needs are critical to successful discharge planning and reducing the likelihood of avoidable readmissions - Screening early in the admission process provides additional lead time for inpatient social workers and others to address needs that need to be addressed prior to discharge, also to coordinate with outpatient social work about needs requiring follow-up after discharge |
|  | Expanding to ED settings | 2022  (scale up phase – in progress) | Planned/ proactive | ACORN Team (informed by interested expressed by local ED departments) | Unit-level | Substitution (where administered) | **Goal:** increase reach; increase health equity  **Contextual factors:**   - ED setting may present a unique opportunity to screen patients who might not otherwise present for outpatient care - Addressing unmet social needs is critical to reducing the likelihood of avoidably presenting to the ED and/or needing to be admitted for an ambulatory care sensitive condition - Screening allows ED social workers to take steps towards addressing immediate needs prior to discharge and to coordinate with outpatient social work about needs requiring follow-up after discharge |

|  | **What was Modified** | **When** | **Planned/ Unplanned** | **Who Decided** | **Level of Delivery** | **Nature of Modifications** | **Reasons** |
| --- | --- | --- | --- | --- | --- | --- | --- |
| **Adaptome - Target Audience Adaptations** Focused on tailoring ACORN to specific patient populations and clinical teams | | | | | | | |
| *Expanded POPULATIONS to whom ACORN is administered* | | | | | | | |
| ***Context***  ***(Population)*** | Adapted and implemented ACORN in a VA primary care clinic specifically serving Veterans experiencing homelessness (VEH) as well as a VA-sponsored tent community  **Content:** tailored screening questions (e.g., housing screen) and process for clinic workflow | 2021  (implementation phase) | Planned/ proactive | ACORN Team along with researchers and clinical partners at a VA primary care clinic for VEH | Patient-level | Tailoring | **Goal:** Increase reach; increase clinical effectiveness; increase appropriateness and acceptability; increase health equity  **Contextual factors:**   - VEH are at high risk for experiencing other social risks and have may have unique contextual factors that need to be taken into account such as lack of access to a place to shower, do laundry, or cook/store food - VEH often require more intensive case management to address unmet needs - Although many, but not all, VEH receiving care at the VA have a caseworker, ACORN offers the opportunity to broadly screen for social risks on a regular, systematic basis |
|  | Currently adapting and implementing ACORN in a Geriatrics clinic  **Content:** currently developing additional tailored screening questions for older adults that are an optional add-on to the core ACORN screener | 2022  (scale up phase – in progress) | Planned/ proactive | ACORN Team along with clinical and VA operations partners | Patient-level | Tailoring; Adding elements | **Goal:** Increase reach; increase clinical effectiveness; increase appropriateness and acceptability; increase health equity  **Contextual factors:**   - Older adults are at increased risk for social risks such as social isolation or lack of transportation, and may have contextual factors including cognitive impairment or functional limitations that uniquely impact other social risks such as food insecurity - Need to identify certain social risks faced by older adults that are less relevant/ generalizable for the overall Veteran population such as elder abuse or neglect and caregiver needs - Older adults may experience barriers to accessing online resources because of lack of access and/or low technology literacy; they may have unique resources available to them such as senior centers or benefits only available to those over age 65 |
|  | **What was Modified** | **When** | **Planned/ Unplanned** | **Who Decided** | **Level of Delivery** | **Nature of Modifications** | **Reasons** |
|  | Expanded ACORN to clinics specifically serving women Veterans | 2018  (pilot phase)  2022  (scale up phase – in progress; adding additional focus on prenatal patients) | Planned/ proactive | ACORN Team along with local VISN leadership  ACORN Team along with local women’s health specialty clinic | Patient-level/  clinic-level | Tailoring | **Goal:** Increase reach; increase health equity  **Contextual factors:**   - Women Veterans are at increased risk for social risks including intimate partner violence and food insecurity^3,4^ - Many women Veterans prefer to receive care in a dedicated women’s health clinic (e.g., due to history of trauma) and may not be reached by screening conducted in general primary care - Women Veterans, and particularly pregnant Veterans, may face unique barriers to accessing resources and may also have unique resources available to them |
| **Adaptome - Mode of Delivery Adaptations** Allowed ACORN to be implemented through multiple modalities for flexibility across sites/contexts | | | | | | | |
| *Expanded HOW the ACORN screener is delivered* | | | | | | | |
| ***Context***  ***(Format)*** | Switched from Veteran self-administered screening to staff-administered screening; Developed EHR template allowing staff to administer ACORN and enter directly in the EHR | 2020 at onset of COVID-19 pandemic  (pilot phase) | Unplanned/ reactive | ACORN team and frontline nursing staff at pilot sites | Clinic-level | Substitution (how administered) | **Goal:** Increase reach; increase health equity  **Contextual Factors:**   - Dramatic increase in telehealth, temporary halt to most in-person visits - Concerns for infection risk with shared electronic tablets in waiting rooms - Switching to staff-administered screening directly in the EHR allowed for delivery of ACORN during virtual visits (phone or video) |
|  | **What was Modified** | **When** | **Planned/ Unplanned** | **Who Decided** | **Level of Delivery** | **Nature of Modifications** | **Reasons** |
|  | Expanding from individual visits to group visits | 2022  (scale up phase – in progress) | Planned/ proactive | ACORN Team with input from VA operations partners | Clinic-level | Substitution (how administered) | **Goal:** Increase reach; increase health equity  **Contextual Factors:**   - Expanding ACORN to group visit settings has the potential to increase the number of Veterans exposed to ACORN - Certain group visits such as advance care planning groups and “whole health” groups lend themselves particularly well to introducing the concept of social risks/social needs as part of the group curriculum, followed by individual Veteran self-administration of ACORN screener |
| *Modified how ACORN screening data recorded and tracked* | | | | | | | |
| ***Context***  ***(Format)*** | Modified how screening data was captured and recorded by linking screening responses in EHR template to structured data fields in EHR | 2020 at onset of COVID-19 pandemic, concurrent with development of EHR template  (pilot phase) | Planned/ proactive | ACORN Team | Organization-level | Tailoring | **Goal:** improve sustainability; facilitate evaluation efforts  **Contextual Factors:**   - Allows screening data to be more easily retrieved and analyzed |
| *Developed model for creation and dissemination of ACORN resource guides* | | | | | | | |
| ***Content*** | Formalized process for creation of geographically-tailored resource guides | 2019  (pilot phase) | Planned/ proactive | ACORN Team | Organization-level | Refining | **Goal:** increase effectiveness; improve sustainability; increase health equity  **Contextual Factors:**   - Allows for greater dissemination and uptake of geographically-tailored guides - Streamlines process for creating guides that align with local needs and contexts and minimizes redundancy in developing tailored resource guides in new settings |
|  | **What was Modified** | **When** | **Planned/ Unplanned** | **Who Decided** | **Level of Delivery** | **Nature of Modifications** | **Reasons** |
| **Adaptome - Cultural Adaptations** Made in response to shifting needs, priorities, and preferences | | | | | | | |
| *Modified ACORN screening questions and/or domains* | | | | | | | |
| ***Content*** | Modified screening questions based on cognitive interviewing and field testing | 2018  (pilot phase) | Planned/ proactive | ACORN Team, based on Veteran and staff feedback | Organization-level | Refining | **Goal:** Increase clinical effectiveness; increase appropriateness and acceptability; increase health equity  **Contextual Factors:**   - Received feedback from Veterans during cognitive testing and follow up interviews around ways to improve clarity and appropriateness of certain questions - Received feedback from staff regarding need to revise interpersonal violence question due to high false positive rates |
|  | Added new screening domain to core screener related to technology, phone, and internet access | 2021  (implementation phase) | Planned/ proactive | ACORN Team and VA operations partners | Organization-level | Adding elements | **Goal:** increase clinical effectiveness; increase appropriateness; increase health equity  **Contextual Factors:**   - Patients experienced increasing needs for technology access during pandemic, particularly due to increased use of virtual care (telehealth) - Availability of VA resources to address digital needs expanded rapidly during the pandemic - Systematic screening for digital needs provides an opportunity to match those with needs to the resources available  (e.g., video-capable device) |
|  | **What was Modified** | **When** | **Planned/ Unplanned** | **Who Decided** | **Level of Delivery** | **Nature of Modifications** | **Reasons** |
|  | Removed domain on interpersonal violence | 2021  (implementation phase) | Planned/ proactive | ACORN Team and National Social Work Program | Organization-level/ facility-level | Removing elements | **Goal:** increase appropriateness  **Contextual Factors:**   - Concern that in some settings, there may not be adequate follow-up or resources available at the time of screening if a safety concern is disclosed - One site elected to use this modified version because they felt interpersonal violence domain was redundant with another screener administered locally at that facility |
|  | Added disposition section to document actions taken in response to identified needs | 2021 (implementation phase) | Planned/ proactive | ACORN Team, Peers, National Social Work Program | Organization-level | Adding elements | **Goal:** increase effectiveness; increase fidelity; assess impact  **Contextual Factors:**   - Tracking actions taken to address needs (e.g., resources and referrals provided) is critical to assess impact and fidelity - List of potential actions may help prompt staff using ACORN to offer interventions - Initially tailored disposition items to social workers and Peers, but recognized need to reconcile a uniform disposition section for dissemination; other/free text fields included for flexibility and clinical utility |
| *Modified presentation of ACORN screener* | | | | | | | |
| ***Context***  ***(Format)*** | Changed the layout of the paper screener | 2022  (scale up phase) | Planned/ proactive | ACORN Team based on feedback from frontline clinical staff | Organization-level | Changes to materials | **Goal:** increase adoption; increase feasibility and acceptability; improve fidelity  **Contextual Factors:**   - Staff expressed need to more easily visually scan paper screener for positive responses |
|  | **What was Modified** | **When** | **Planned/ Unplanned** | **Who Decided** | **Level of Delivery** | **Nature of Modifications** | **Reasons** |
|  | Modified the layout of the EHR template | 2022  (scale up phase) | Planned/ proactive | ACORN Team, based on feedback from frontline clinical staff | Organization-level | Changes to materials | **Goal:** increase adoption; increase feasibility and acceptability; improve fidelity  **Contextual Factors:**   - Staff requested the addition of free text fields to the EHR template to increase clinical utility of the template during encounters |
| *Mapped ACORN elements onto existing screening workflows* | | | | | | | |
| ***Context***  ***(Format)*** | Aligned data capture for food and housing questions with existing VA screening for these domains | 2020 at onset of COVID-19 pandemic, concurrent with development of EHR template  (pilot phase) | Planned/ proactive | ACORN Team | Organization-level | Tailoring | **Goal:**  increase adoption; increase acceptability; increase fidelity; increase sustainability  **Contextual Factors:**   - Allows for concurrent completion of ACORN and existing VA screening requirements for food insecurity and housing instability - Streamlines workflows and minimizes redundancy of screening efforts - Increases staff buy-in by satisfying required screening for food and housing |

^1^ Wiltsey SS, Baumann AA, Miller CJ. The FRAME: an expanded framework for reporting adaptations and modifications to evidence-based interventions. Implement Sci. 2019;14(1):58.

^2^ Chambers DA, Norton WE. The Adaptome: advancing the science of intervention adaptation. Am J Prev Med. 2016 Oct;51(4S2):S124-131.

^3^ Dichter ME., Haywood TN, Butler AE, Bellamy SL, Iverson KM. Intimate Partner Violence Screening in the Veterans Health Administration: Demographic and Military Service Characteristics. Am J Prev Med 52, no. 6 (June 2017): 761–68.

^4^ Cohen AJ, Dosa DM, Rudolph JL, Halladay CW, Heisler M, Thomas KS. Risk Factors for Veteran Food Insecurity: Findings from a National US Department of Veterans Affairs Food Insecurity Screener. Public Health Nutr, November 8, 2021, 1–26.

**VISN**: Veterans Integrated Service Network (regional VA Healthcare System); **PACT**: Patient-aligned care teams; **VEH**: Veterans experiencing homelessness; **EHR**: Electronic health record
